# Prädiktion der pathologischen Komplettremission beim Ösophaguskarzinom nach neoadjuvanter Radiochemotherapie mittels FDG-PET/CT und diffusionsgewichteter MRT

**DOI:** 10.1007/s00066-021-01813-1

**Published:** 2021-07-19

**Authors:** Markus Hecht, Sabine Semrau

**Affiliations:** Universitäts-Strahlenklinik Erlangen, Friedrich-Alexander-Universität Erlangen-Nürnberg, Universitätsstraße 27, 91054 Erlangen, Deutschland

## Hintergrund und Ziele

Nach neoadjuvanter Radiochemotherapie (RCT) von Ösophaguskarzinomen erreicht eine relevante Patientenzahl eine pathologische Komplettremission. Eine exakte Identifikation von Komplettremissionen könnte eine Patientengruppe identifizieren, bei denen weder eine zusätzliche Operation noch eine Dosiserhöhung der Strahlentherapie (RT) auf über 50 Gy hinaus erforderlich ist. Damit könnte ähnlich wie beim Rektumkarzinom in dieser Subgruppe ein kompletter Organerhalt durch RCT erreicht werden und die Therapieletalität im Zusammenhang mit der Resektion vermieden werden; diese liegt selbst bei erfahren Zentren mit vielen Resektionen in Deutschland bei 7 %. Ziel der hier kommentierten, prospektiven multizentrischen Studie war es, durch multimodale Bildgebung mittels ^18^F‑FDG-PET/CT und einer diffusionsgewichteten MRT pathologische Komplettremissionen präoperativ zuverlässig voraussagen zu können.

## Patienten und Methoden

Patienten mit Erstdiagnose eines nichtmetastasierten Ösophaguskarzinoms erhielten eine neoadjuvante RCT. An den beiden niederländischen Zentren wurde diese in Einzeldosen von 1,8 Gy bis zu einer Gesamtdosis von 41,4 Gy mit simultanem Carboplatin/Paclitaxel durchgeführt. Am MD Anderson Cancer Center erhielten die Patienten eine Gesamtdosis von 50,4 Gy in Einzeldosen von 1,8 Gy zusammen mit einer simultanen Chemotherapie bestehend aus 5‑Flurouracil in Kombination mit Taxanen oder Platinsubstanzen. Im Anschluss erhielten alle Patienten eine transhiatale oder transthorakale Ösophagektomie mit Lymphadenektomie und Rekonstruktion. Die Bildgebung mittels ^18^F‑FDG-PET/CT und diffusionsgewichteter MRT erfolgte vor Beginn der RCT, während der RCT (im Median 13 Tage nach Beginn) und 5 Wochen nach Abschluss. Die in der ^18^F‑FDG-PET/CT erfassten Parameter beinhalteten den mittleren und maximalen „standardized uptake value“ (SUV_mean_, SUV_max_), das „metabolic tumor volume“, die „total lesion glycolysis“ (TLG) und das Produkt aus „metabolic tumor volume“ und SUV_max_. In der diffusionsgewichteten MRT wurden die mittleren „apparent diffusion coeffizient“-Werte (ADC) ermittelt. Diese wurden jeweils im Verhältnis zum Ausgangswert betrachtet. Aus diesen Werten wurde zusammen mit der Histologie des Tumors ein Modell zur Prädiktion der erwarteten pathohistologischen Komplettremission errechnet.

## Ergebnisse

Zwischen Oktober 2013 und Juli 2017 wurden 82 Patienten in den 3 Zentren eingeschlossen, wovon allerdings nur die Daten von 69 Patienten analysiert werden konnten. Histologisch handelte es sich vor allem um Adenokarzinome (*n* = 57, = 83 %). Bei 18 Patienten (26 %) wurde eine pathologische Komplettremission erreicht, wobei diese signifikant häufiger bei Patienten mit Plattenepithelkarziomen auftrat. In der FDG-PET/CT nach RCT unterschieden sich die Parameter SUV_mean_ und TLG im Verhältnis zum Ausgangswert in den Gruppen mit und ohne Komplettremission. In der diffusionsgewichteten MRT während RCT unterschied sich der ADC im Verhältnis zum Ausgangswert in den Gruppen mit und ohne Komplettremission. Am besten geeignet zur Vorhersage der pathologischen Komplettremission war ein Modell, in das die Histologie, der ADC in der MRT während Therapie im Verhältnis zum Ausgangswert und der SUV_mean_ im FDG-PET/CT nach Therapie im Verhältnis zum Ausgangswert eingingen.

## Schlussfolgerung der Autoren

Veränderungen im ^18^F‑FDG-PET/CT nach Therapie und in der diffusionsgewichteten MRT während der RCT können zur Prädiktion von pathologischen Komplettremissionen bei Patienten mit Ösophaguskarzinom herangezogen werden. Die Ergebnisse beider Untersuchungen sind dafür vermutlich komplementär.

## Kommentar

Die CROSS-Studie hat bereits den Nutzen einer neoadjuvanten Radiochemotherapie bei resektablen Ösophaguskarzinomen bezüglich des Überlebens (OS) der Patienten belegt. Darüber hinaus zeigte sich, dass 49 % der Patienten mit Plattenepithel- und 23 % der Patienten mit Adenokarzinom eine komplette Tumorremission im Resektionspräparat aufwiesen [1]. Das heute gültige Behandlungskonzept besagt deshalb, dass bei operablen Tumoren nach der neoadjuvanten RCT immer eine kurativ intendierte Resektionsbehandlung zu erfolgen hat. Das operative Vorgehen birgt allerdings eine Reihe von Risiken, wie Anastomoseninsuffizienz, Blutungen, ARDS, Chylusfisteln oder Nervenverletzungen [2]. Gerade beim Vorliegen von Ösophaguskarzinomen und insbesondere bei Plattenepithelkarzinomen haben die Patienten hochfrequent Begleiterkrankungen, die zumindest postoperative Komplikation implizieren können, die wiederrum mit einem Anstieg der Letalität auf 12 % (in Häusern mit hohen Fallzahlen) bzw. 20 % (bei Häusern mit niedrigen Fallzahlen) auch in Deutschland einhergehen. Zudem erfahren resezierte Patienten Einbußen an Lebensqualität; diese nähert sich erst nach etwa 2 Jahren derjenigen von Patienten nach alleiniger RCT, also ohne Operation, an [3]. Daher stellt sich beim Ösophaguskarzinom noch dringlicher als bei Vorliegen eines Rektumkarzinoms die Frage, ob die Subgruppe der Patienten mit Komplettremissionen nach neoadjuvanter RCT sicher identifiziert werden kann, um eine Operation zu vermeiden. Uns scheint es dabei sogar gerechtfertigt, eine geringe Rate an rezidivierenden Patienten in Kauf zu nehmen, bei denen sich trotz attestierter Komplettremission nach RCT ein Lokalrezidiv einstellt. Diese können nämlich noch einer Salvageresektion nach 41,4 Gy bzw. 50,4 Gy zugeführt werden.

Mehrere Strategien zur Identifizierung von kompletten Respondern nach neoadjuvanter RCT werden insbesondere von niederländischen Arbeitsgruppen und der RTOG vorangetrieben. Während die RTOG in der RTOG 0246-Studie auf radiologische Bildgebung, Endoskopie und Abwarten setzte, um den Grad der Remission herauszufinden [4], befassen sich niederländische Arbeitsgruppen mit multimodalen diagnostischen Konzepten zur frühen Identifikation solcher Verläufe. Eines ist das Konzept der perSANO-Studie, bei der strategische und geschichtete Biopsien („bite-on-bite biopsy“) und die Endosonographie im Vordergrund stehen [5]. Die andere ist das der PET- und MRT-Bildgebung. Die hier besprochene Studie zur multiparametrischen Bildgebung mittels ^18^F‑FDG-PET/CT und der diffusionsgewichteten MRT sind erste Ansätze dazu.

Bisherige Studien haben zunächst meist nur eine bildgebende Modalität untersucht. In einer Metaanalyse zur FDG-PET/CT wurde der Rückgang des SUV_max_ 2 Wochen nach Beginn der RT gefunden als geeigneter prädiktiver Parameter für den Endpunkt „< 10 % lebende Tumorzellen“. Jedoch war die Sensitivität zu gering, ebenso die Spezifität, um den Einsatz in der Klinik zu wagen [6]. Zudem wurden erste Daten zur Wertigkeit der diffusionsgewichteten MRT in dieser Situation veröffentlicht [7]. Die komplementäre Bildgebung mit beiden Methoden, also die FDG-PET/CT nach Therapie, die relative SUV_mean_, die TLG sowie die diffusionsgewichtete MRT während der RCT und auch der relative ADC-Wert zu verschiedenen Zeitpunkten der Therapie, haben in der hier diskutierten Publikation die zuverlässigsten prädiktiven Parameter identifiziert für die Vorhersage pathohistologischer Tumor-Komplettremissionen. Zudem entwickelten die Autoren einen Score, der auch die Tumorhistologie berücksichtigt und so die pathohistologische Komplettremission relativ präzise (Sensitivität von 83 %) im untersuchten Kollektiv vorhersagen konnte. Auf diese Entwicklung können zukünftig personalisierte Therapiekonzepte aufgebaut werden.

Allerdings weist die Studie, wie andere in der Methodenentwicklung übrigens häufig auch, mehrere Schwachpunkte auf, so dass man das hier beschriebene Konzept keineswegs schon in der klinischen Routine etablieren kann. Neben methodischen Problemen, dass beispielsweise nur 69 der 82 eingeschossenen Patienten analysiert werden konnten, was u. a. an den zu kleinen Tumorvolumina und fehlenden Signalen in der Bildgebung lag, wurden die Patienten auch nicht konzeptionell einheitlich behandelt. Die Gesamtdosis der Bestrahlung betrug entweder 41,4 Gy oder 50,4 Gy, was relativ betrachtet eine mehr als 20 % höhere Dosis darstellt. Zudem erfolgte die simultane Chemotherapie entweder mit Carboplatin/Paclitaxel oder 5‑Flurouracil-basiert (zusammen mit Platin oder Taxan). Die größte Schwäche besteht allerdings darin, dass lediglich eine explorative Kohorte untersucht wurde und die so generierten Daten bisher nicht an anderen Patientenkollektiven validiert werden konnten. Daher besteht ein relevantes Risiko, dass die Etablierung des Modells durch „overfitting“ im verwendeten Patientenkollektiv deutlich bessere Ergebnisse geliefert hat, als es der Realiltät entspricht.

Nichtsdestotrotz scheinen beide bildgebenden Verfahren einen gewissen Wert für die Prädiktion des Therapieansprechens auf eine neoadjuvante RCT von Ösophaguskarzinomen zu besitzen. Sicher ist aber auch, dass es noch kein perfektes Modell für ihren Einsatz gibt. An ihm muss noch gearbeitet werden. Ein Anfang ist durch die niederländische Arbeitsgruppe gemacht. Man darf auf die Phase-III-Studien gespannt sein, die das CROSS-Protokoll inklusive der frühen Resektion bei klinisch als Komplettresponder eingeschätzten Patienten mit denen des Zuwartens und Salvageresektion vergleicht. Bereits in der Studie von Bedenne und Mitarbeitern [8] zeigte sich nämlich, dass die Responder nach RCT, die mithilfe des *altertümlichen* Röntgenbreischluckes erkannt worden waren, keinen Überlebensnachteil dadurch hatten, dass auf die Resektion zugunsten einer Fortführung der RCT verzichtet wurde.

## Fazit

Die Daten dieser Studie legen einen prädiktiven Wert der diffusionsgewichteten MRT während einer neoadjuvanten Radiochemotherapie und der ^18^F‑FDG-PET/CT nach neoadjuvanter Radiochemotherapie hinsichtlich pathohistologischer Komplettremissionen von Ösophaguskarzinomen nahe. Die Ergebnisse mit Berechnung eines prädiktiven Scores müssen als explorativ eingestuft werden und bedürfen der Validierung durch Nachfolgeprotokolle. Zukünftig könnten diese Verfahren zur Personalisierung der Therapie von Patienten mit Ösophaguskarzinom beitragen.

*Markus Hecht und Sabine Semrau, Erlangen*
